# Editorial: Molecular Intricacies of *Trichoderma*-Plant-Pathogen Interactions

**DOI:** 10.3389/ffunb.2022.892228

**Published:** 2022-04-27

**Authors:** Prasun K. Mukherjee, Benjamin A. Horwitz, Francesco Vinale, Pierre Hohmann, Lea Atanasova, Artemio Mendoza-Mendoza

**Affiliations:** ^1^Nuclear Agriculture and Biotechnology Division, Bhabha Atomic Research Centre, Mumbai, India; ^2^Faculty of Biology, The Technion-Israel Institute of Technology, Haifa, Israel; ^3^BAT Center—Interuniversity Center for Studies on Bioinspired Agro-Environmental Technology, University of Naples Federico II, Portici, Italy; ^4^Department of Veterinary Medicine and Animal Productions, University of Naples Federico II, Naples, Italy; ^5^Sustainable Plant Protection Programme, IRTA Institute of Agrifood Research and Technology, Lleida, Spain; ^6^Institute of Food Technology, University of Natural Resources and Life Sciences, Vienna (BOKU), Vienna, Austria; ^7^Department of Wine, Food and Molecular Biosciences, Lincoln University, Canterbury, New Zealand

**Keywords:** *Trichoderma*, antagonism, symbiosis, biocontrol, secondary metabolism

*Trichoderma* spp. are widely used as plant disease biocontrol agents around the world (Guzmán-Guzmán, 2019). Initial research on *Trichoderma* focused on their biocontrol ability mediated by mycoparasitism and antibiosis. *Trichoderma* spp. are known for direct antagonistic action on plant pathogenic fungi, and more recently for indirect suppression *via* induced defense. Thus, with the discovery that *Trichoderma* spp. can internally colonize roots and invoke defense responses in plants, attention of the research community largely shifted toward understanding how the plants and *Trichoderma* communicate with each other leading to a symbiosis-like relationship (Harman et al., 2004; Hohmann, 2012). At the initial attachment stage, *Trichoderma* spp. are known to secrete hydrophobins that could aid in adhesion to the root surface (Viterbo and Chet, 2006). Following attachment, secretion of plant cell wall degrading enzymes like pectate lyase (Morán Diez et al., 2009) and swollenin (Brotman et al., 2008; Andberg et al., 2015; Cosgrove, 2017) could facilitate penetration. It is important to note that penetration into the root is limited, and indeed genes encoding plant cell wall degrading enyzymes are downregulated early in root colonization (Estrada-Rivera et al., 2020). This occurs along with high representation of glycosyl hydrolases in the secretome (Nogueira-Lopez et al., 2018). Soluble enzymes may still be present in the secretome after the corresponding transcripts decrease, highlighting the need for obtaining well-resolved time course experiments for both gene expression and protein abundance during early colonization events. *Trichoderma* fungi are known to secrete a large number of small secreted cysteine-rich proteins (SSCPs) that might be involved in modulation of plant defense, fine tuning of which may be responsible for the outcome of this association. Though not clearly established, it seems possible that *Trichoderma* SSCPs initially suppress plant defense, and once the colonization is complete, induces plant defense to enter into a symbiosis-like relationship. Unlike mycorrhiza, there seems to exist no specificity in *Trichoderma*-plant association, which appears to be quite universal. For example, AM mycorrhizal fungi cannot colonize cruciferous roots exhibiting a level of specificity, but *Trichoderma* can effectively colonize such roots, indicating a generalist type of lifestyle.

Although priming of plant immunity through root interactions contributes to plant protection, the role of mycoparasitism cannot be underestimated, as it directly kills the pathogens reducing the inoculum load (Druzhinina et al., 2011; Mukherjee et al., 2022). *Trichoderma* secondary metabolite production is equally important for both host-*Trichoderma* communication, e.g., modulating the plant metabolism (Vinale and Sivasithamparam, 2020; Zhang et al., 2021), and antibiosis leading to competitive exclusion of the pathogen or other microbes. This helps it to establish in a particular niche like the rhizosphere (Contreras-Cornejo et al., 2016). Some strains are also nematicidal that can kill juveniles and eggs (Poveda et al., 2020). In addition, evidences are emerging on *Trichoderma*-mediated control of insect pests (Poveda, 2021) and viruses (Vitti et al., 2016). *Trichoderma* spp. also have potential applications in bioremediation of xenobiotics and heavy metals (Tripathi et al., 2013). Even though individual gene studies had already started to shed light on the molecular mechanisms of various aspects of *Trichoderma* biology, including mycoparasitism, secondary metabolism and induced resistance, availability of the ‘omics data is already shaping the future of *Trichoderma* research (Kubicek et al., 2011; Schmoll et al., 2016; Schmoll and Zeilinger, 2021). For instance, more than a dozen species have been sequenced and the whole-genome sequences of several strains are available in the public database. It is to be seen how this rapid progress matches with the physiological studies leading to a better understanding of *Trichoderma* biology. Our attempt here has been to set the stage for such research through a compilation of articles covering various aspects of how *Trichoderma* interacts with plants and pathogens at the molecular level. This collection harbors two review articles that give an overview of the developments in the field and three research papers.

In *Trichoderma*-plant interactions, fungal cues that lead to the internalization of the biocontrol agent and induction of defense signaling have received much attention. The article of Leibman-Markus et al. addresses this aspect from the plant side. It was already known that tomato plants harbor a decoy receptor LeEIX1 (belonging to leucine-rich repeat cell-surface glycoproteins) that negatively affects induced defense response by binding to the *Trichoderma harzianum* ethylene inducing xylanase (an elicitor). The authors used the CRISPR/CAS9 technology to mutate LeEIX1, thus promoting binding of the elicitor with LeEIX2, leading to enhanced induced resistance. This article thus not only helps in understanding the molecular intricacies of *Trichoderma*-plant interactions, but also shows that the host plant can be modified to improve the performance of *Trichoderma* spp. as biocontrol agents.

The diversity of natural products produced by members of the genus *Trichoderma* is vast, with an enormous potential across diverse industry sectors, including agriculture, medicine, and food. The *Trichoderma* genus is widely used as a biological control against diverse microorganisms, as well as biofertilizers and biostimulants of important commercial crops. The market for biological products, including biofertilizers, biological control and biostimulants is expected to reach 5 billion US dollars by 2026. The publicly available genomes open up great opportunities for entrepreneurs to build multimillion-dollar companies across the industry sector. The review by Rush et al. suggests integrating various “omics” technologies, next-generation biodesign, machine learning, and artificial intelligence approaches to significantly advance bioprospecting goals. The authors propose a roadmap for assessing the potential impact of already known or newly discovered *Trichoderma* species for biocontrol applications. The authors suggest using free access tools to screen the publicly available *Trichoderma* genome sequences for identifying the prevalence of potential biosynthetic gene clusters for secondary metabolites and antimicrobial peptides in genomes. This is considered as an initial step toward predicting which organisms could increase the diversity of bioactive natural products. Moreover, this article discusses the possible high-throughput strategies for screening organisms to discover and characterize new natural compounds and how these findings could have a bearing on both fundamental and applied research fields.

The gene expression signatures of both plant and fungal partners give insight into how *Trichoderma* and host respond to each other at the transcriptome (Chacón et al., 2007; Rubio et al., 2012; Malinich et al., 2019) and proteome (Nogueira-Lopez et al., 2018) levels. The earliest changes in gene expression are relevant to the first steps of the molecular dialogue. The study by Taylor et al. focused on such early changes and used co-expression network calculations to identify target genes occupying central positions in the network. Of 18 genes chosen in this way, six are small secreted cysteine-rich proteins, including Sm2, a known effector of plant systemic resistance (Gaderer et al., 2015). These, and other candidates like one annotated as a bicupin found in the maize apoplast (Nogueira-Lopez et al., 2018), will be promising for detailed genetic studies. The pipeline was used to compare response of the wild type to that of mutants in two genes encoding small secreted proteins, Sm1 and Sir1. These two small secreted proteins balance activation and suppression of *Trichoderma*-induced plant immunity (Wang et al., 2020). Intriguingly, Sir1, not Sm1, appears as a major regulator of *Trichoderma* gene expression in response to maize interaction.

Nanotechnology presents a promising research area to reduce the application of conventional pesticides and fertilizers in agriculture. Bacteria, fungi and plants are used in biogenic synthesis of nanoparticles (NPs) to extract and isolate reducing agents and stabilizers. *Trichoderma* is, besides *Fusarium, Aspergillus*, and *Penicillium*, one of the main fungal genera used for the mycosynthesis of NPs. Ramírez-Valdespino and Orrantia-Borunda reviewed recent efforts in *Trichoderma*-mediated mycosynthesis of NPs, with a summary of biosyntheses, characteristics and antimicrobial activities of silver, selenium, gold, copper, and zinc NPs. Furthermore, the authors reviewed the applicability and the antifungal effects of different *Trichoderma* metal NPs on plant pathogenic fungi and, finally, the tolerance mechanisms that *Trichoderma* itself exhibits against such metal NPs. Summarized, *Trichoderma* appears to be a valuable mediator for the biosynthesis of NPs, which can be used for the formulation of agrochemicals and the treatment of plant and human pathogens.

Quinoa may have been domesticated as a crop thousands of years ago by the indigenous people of the Andean Altiplano, but it is only recently attracting interest because of its nutritional value and resistance to abiotic stresses. As for other crops, *Trichoderma* can improve quinoa yield. Therefore, the *Trichoderma*-quinoa interaction was studied by Rollano-Peñaloza et al. at the transcriptomic level. The two quinoa cultivars studied showed growth inhibition rather than promotion when grown *in vitro* for extended times in interaction with *T. harzianum* and *T. afroharzianum*. Considering these novel plant-*Trichoderma* interacting pairs, the authors observed induction of quinoa defense genes in a cultivar-specific manner. Furthermore, a clade of genes for germin-like proteins (GLP), important in oxidative stress response and for protection from superoxide toxicity, was induced more strongly in one cultivar. Thus, these transcriptomic findings touch on the diversity of *Trichoderma*-plant interactions, relevant to both farming and plant breeding.

*Trichoderma* spp. have become an integral part of agricultural systems worldwide. Being broad spectrum in action and largely crop non-specific, these beneficial plant fungi are widely used across almost all crops. Understanding the molecular intricacies of *Trichoderma*-plant-pathogen interactions helps to improve the efficacy of commercial applications. With the idea to shed light on recent developments in understanding the molecular mechanisms of biocontrol and plant growth promotion, this Research Topic was conceived. The Editors are thankful to the authors who submitted quality articles for this Research Topic on molecular intricacies of *Trichoderma*. Their contributions point toward important trends in this area of research and will help establish this new Frontiers journal that brings together the various fields of fungal biology.

## Author Contributions

All authors listed have made a substantial, direct, and intellectual contribution to the work and approved it for publication.

## Conflict of Interest

The authors declare that the research was conducted in the absence of any commercial or financial relationships that could be construed as a potential conflict of interest.

## Publisher's Note

All claims expressed in this article are solely those of the authors and do not necessarily represent those of their affiliated organizations, or those of the publisher, the editors and the reviewers. Any product that may be evaluated in this article, or claim that may be made by its manufacturer, is not guaranteed or endorsed by the publisher.
